# Prediction of Ultimate Strain for Rectangular Reinforced Concrete Columns Confined with Fiber Reinforced Polymers under Cyclic Axial Compression

**DOI:** 10.3390/polym12112691

**Published:** 2020-11-15

**Authors:** Theodora D. Fanaradelli, Theodoros C. Rousakis

**Affiliations:** Laboratory of Reinforced Concrete and Seismic Design of Structures, Department of Civil Engineering, Faculty of Engineering, Democritus University of Thrace (D.U.Th.), 67100 Xanthi, Greece; tfanarad@civil.duth.gr

**Keywords:** FRP, confinement, modeling, rectangular reinforced concrete, strain, FEM

## Abstract

This paper investigates the crucial design parameters for the prediction of the ultimate axial compressive deformation of reinforced concrete columns externally confined with fiber reinforced polymer (FRP) materials. Numerous test results of available columns with a square and rectangular section under cyclic axial loading were gathered in an advanced database. Herein, the database is enriched with necessary design parameters in order to address the unique tensile strain field variation of the FRP jacket. Since there is a lack of consequent recording of the FRP strain field in existing experiments, three dimensional pseudodynamic finite element analyses results from several characteristic cases of tested columns are utilized to address this gap. Therefore, a hybrid experimental–analytical database is formed, including several critical FRP strains, steel strains and deformations. A modified model is proposed to predict the ultimate axial strain for reinforced concrete columns externally confined with FRP materials. The proposed model aims to address indirectly the effects of the internal steel cage, concrete section shape and of their interaction with the external FRP jacket on the critical tensile strain of the FRP jacket at failure of the column. The predictive performance of the model over the available tests of (reinforced concrete) RC columns under cyclic compression is remarkably improved when compared against the performance of other existing models. It provides predictions with average ratio (AR) of 0.96 and average absolute error (AAE) of 36.5% and therefore may contribute to safer seismic resistant redesign.

## 1. Introduction

The use of fiber reinforced polymers (FRPs) has been widely accepted as a successful confinement technique for existing reinforced concrete (RC) columns in buildings and bridges. The benefits of this technique are the improvement of the axial strength, strain ductility and energy dissipation capacity of the confined concrete. Thus, the behavior of concrete columns confined with FRP has been extensively studied, leading to a significant number of stress–strain models, some focused on circular cross-sections [1,2,3,4,5] and others focused on square and rectangular ones [6,7,8,9,10,11,12,13,14,15,16,17]. In addition to that, some studies are concentrated on modeling the stress–strain behavior of both circular and non-circular FRP confined concrete columns [17,18,19,20,21,22]. Other studies model the overall stress–strain curves of columns under cyclic axial loading [23,24]. These models are mostly design-oriented models [6], based on empirical or semiempirical relationships. They take into account parameters that are easily understood and available to designers, such as the corner radius, the stiffness of the FRP and concrete strength. Recently, Zeng et al. [25] compared the performance of five representative stress–strain models [6,14,15,16,21] for FRP-confined RC concrete columns against the experimental behavior of nine large scale columns under monotonic axial loading. They concluded that Cao et al.’s [21] model best predicts the ultimate axial stresses of the nine large scale columns, and the one by Lam and Teng [6]. All above mentioned models underestimated the ultimate axial strains of the tested columns.

There are also analysis-oriented thorough studies of the behavior of the confined columns and provide the stress–strain curves using closed-form equations. Recently, Lin and Teng [26] proposed a model for the stress–strain behavior of square FRP-confined concrete columns utilizing advanced finite element (FE) simulations. These analyses were conducted to gain an in-depth understanding of the confinement mechanism. The results, combined with the experimental data, were used to formulate a new model. Additionally, earlier Jiang et al. [12] introduced a modified local-to-global methodology to understand the effect of fiber reinforced polymer (FRP) confinement on square concrete section, which led to evaluating the stress–strain behavior of the columns. Megalooikonomou and Papavasileiou [27] also proposed an iterative approach to model both the axial and lateral stress–strain response of square and rectangular FRP-confined RC concrete columns with sparse stirrups, so that the contribution of the stirrups is not taken into account. Except for the studies by Cao et al. [5], Eid and Paultre [9], Isleem et al. [10] and Wang and Isleem [17], which refer to columns with internal steel reinforcement, most of these models do not take into account the presence of the internal steel reinforcement.

Fanaradelli et al. [28,29] investigated all available stress and strain models of the literature against all available test results through an extended database and concluded that with suitable modifications, several existing design models for plain concrete columns of rectangular section confined with FRPs could provide reliably the maximum and ultimate axial stresses for the corresponding concrete columns with internal steel reinforcement, subjected to monotonic or cyclic axial loading. In particular, the assessment of the database proved that the average absolute error (AAE) of axial stress compared to the experimental test results for RC columns under monotonic or cyclic axial loading can easily be improved by adding and considering the full steel bars’ and stirrups’ contribution according to Rousakis and Karabinis [30] or variable contribution of internal steel bars according to Rousakis and Karabinis [31]. The final AAE was around 15%. Numerous studies on the prediction of ultimate axial strains on plain concrete circular concrete columns under monotonic axial loading presented AAEs higher than 25% [32,33,34]. For columns with square and rectangular cross sections under monotonic axial compression the AAE of ultimate axial strain is higher than 30% [6,15,28,34]. For plain non-circular columns under cyclic axial loading the AAE for the prediction of strain at failure is still higher than 30% [29] and for RC rectangular columns the prediction presented AAEs higher than 50% [29]. However, FRP confinement retrofit concern seismic resistant design and therefore available experiments on RC columns under cyclic loading are of great significance. Therefore, this paper focuses on square and rectangular RC concrete columns under cyclic loading that constitute the vast majority of cases in real practice. Furthermore, the prediction of the strain at ultimate seems to require additional critical design parameters that are usually not recorded throughout the available experimental procedures. The accuracy of ultimate axial strain models is of the greatest significance as it affects the success of the performance-based design, rather than the prediction of the column axial strength. That is, the reliable ultimate axial strain of FRP retrofitted RC columns is critical in redesign as it is incorporated in several models at the section or member or structure level, while they are also included in calculations of the performance criteria at the section or member level in seismic resistant redesign. Therefore, the focus of this paper is on the ultimate axial strain of RC columns under cyclic axial loading. Fanaradelli and Rousakis [35] conducted advanced 3-dimensional pseudodynamic FE analyses and investigated the variation of the FRP strain field throughout loading of RC columns for different characteristic tests available in the literature to address missing design parameters. These columns were modeled and analyzed under axial concentric loading–unloading–reloading cycles of increasing compressive deformations. They covered the effects of a wide range of critical parameters of the retrofit of RC columns with FRPs (slenderness of bars, sparse stirrups, steel quality and quantity, type and layers of FRP jacket, type of impregnation resins and reinforcing fibers, corner radius, concrete strength, predamages, etc.). Besides the investigation of the aspects of the main FRP failure criterion, they researched analytically the deformations of the internal steel bars and stirrups and the concrete damage accumulation.

The aim of the present study is to utilize the retrieved values of the variation of the additional critical design parameters and enrich the original experimental database, forming for the first time a hybrid experimental–analytical database for RC columns similar to the one already formed for RC beams strengthened in shear with FRP (Rousakis et al. [36]). The advanced hybrid database of RC columns confined with FRPs includes several critical strain and deformation parameters that indirectly account for the effects of the slenderness of bars, sparse stirrups, steel quality and quantity, type and layers of FRP jacket, corner radius, concrete strength, etc. The retrieved characteristic analytical parameters are utilized to propose suitable modifications of existing design models for the prediction of the ultimate axial strains of such columns. The proposed model is compared against the experimental results of numerous RC columns under cyclic loading and its performance is satisfactory.

## 2. Experimental Database

### 2.1. Square and Rectangular RC FRP—Confined Concrete Columns under Cyclic Axial Loading

In order to check the accuracy of the existing strain models, an experimental database has been formed through an extensive literature research. This database was presented in the previous work by Fanaradelli and Rousakis [29] and includes only the results for FRP-confined concrete columns of square and rectangular cross-sections under cyclic axial loading. This database includes all 44 test results from specimens with internal steel reinforcement from five different studies, taking place between 2008 and 2018. The database includes all the characteristics of the specimens: naming, shape and dimension of the cross-section (h and b are the length of the longer and shorter sides of the column, respectively, H is the height of the specimens and *r_c_* is the corner radius) and the mechanical characteristics of concrete (*f_co_* is the unconfined compressive concrete strength, *f_cc_* and *e_cc_* are the peak axial compressive stress and the corresponding axial strain of FRP-confined concrete, *f_cu_* and *e_cu_* are the ultimate axial compressive stress and the corresponding strain of FRP-confined concrete) and steel materials (*f_y,long_* and *f_y,stirrup_* are the yield strength of longitudinal steel bars and stirrups, respectively; Table 1). The database also contains the material properties of the FRP strengthening (carbon FRP (CFRP) and glass FRP (GFRP)), its detailing, the number of layers, the FRP thickness (*t_FRP_*) and its elastic modulus (*E_FRP_*; Table 1).

The collected experiments included rectangular cross sections (19 specimens) or square sections (25 specimens). All specimens included transverse and longitudinal internal steel reinforcement. Most of the tested columns were strengthened with carbon FRP (CFRP), while only 14 specimens were confined with GFRP jackets. The layers of the FRP materials ranged from 1 to 9 and partial wrapping was not included. The corner radius (*r_c_*) of the specimens varied from 0 to 40 mm, the unconfined compressive concrete strength (*f_co_*) varied from 10.83 to 50 MPa and the nominal yield stress of reinforcement ranged from 200 to 500 MPa. Special care was taken to distinguish the results of 27 specimens having stress–strain curves with descending inelastic (second) branches (i.e., *f_cu_* < 0.85 × *f_cc_* < *f_cc_*) and 17 columns having ascending second branches (i.e., *f_cu_* = *f_cc_*). So, for columns with ascending second branches it was considered that *f_cu_* = *f_cc_* while for columns with descending second branches, if the ultimate stress f*_cu_* was lower than 15% of the maximum strength, there was a revision of this value with the 0.85 × *f_cc_* and of the corresponding value for *ε_cu_* from the experimental stress–strain curves of the test results.

In Fanaradelli and Rousakis [29] the predictive performance of several existing ultimate strain design models, originally proposed for plain rectangular concrete columns confined with FRP materials under monotonic axial loading [16] and for plain [24] and RC [23] rectangular concrete columns under cyclic axial loading, were assessed over the experimental results of RC columns under cyclic axial loading. It was concluded that for columns under cyclic axial loading with internal steel reinforcement, the prediction of ultimate axial strain *ε_cu_* presents low accuracy for all assessed design models [16,23,24].

As the original models did not address the effects of the internal steel reinforcement the AAE was higher than 50%. The model by Hany et al. [24] had AAE 62.8% and AR 0.94 and the model by Wang et al. [23] had AAE 138.9% and AR 2.23. However, it was concluded the model by Wei and Wu [16] may be used for columns with descending second branches as well and has the best overall performance among all investigated models (predicts the strain at failure with average error 56.1% and average ratio 1.14) [29].

### 2.2. Square and Rectangular RC FRP—Confined Concrete Columns under Monotonic Axial Loading

The experimental database by Fanaradelli et al. [28] includes the results for FRP-confined RC concrete columns of square and rectangular cross-sections under monotonic axial loading. The database includes 130 test results collected from 18 different studies, taking place between 2001 and 2018. So, the database includes 29 reinforced concrete columns with rectangular cross-section and 101 with square cross-section. All the specimens in the database are fully wrapped with FRP sheets. There are 87 columns externally reinforced with CFRP, 40 columns with GFRP and 3 with aramid FRP (AFRP). Finally, the layers of the FRP materials ranged from 1 to 9. The corner radius (*r_c_*) of the specimens varied from 0 to 40 mm, the unconfined compressive concrete strength (*f_co_*) varied from 13.0 to 46.3 MPa and the nominal yield stress of reinforcement ranged from 200 to 587 MPa. Finally, the database included 68 specimens having stress–strain curves with descending second branches and 62 columns having ascending second branches. All the characteristics of the specimens were available: shape and dimension of the cross-section, mechanical characteristics of concrete and steel materials, the material properties of the FRP strengthening (elastic modulus), number of layers and the FRP thickness (Table 2). Similar with the results of the database with columns under cyclic axial loading [29], the assessment of the existing models in predicting the ultimate axial strain of concrete was proved to be insufficient [6,7,16,18,41,42,43,44,45] with AAEs higher than 50% [28]. Despite the fact the columns under monotonic loading are not the focus of this research, the available database may be utilized for additional assessments.

## 3. Model by Wei and Wu for Plain FRP—Confined Columns

In what follows, the model by Wei and Wu [16] is used as the basis for suitable modifications and is briefly presented. The model by Wei and Wu [16] was based on a large database including data from 29 published experimental studies on plain concrete columns from the international literature. In particular, the database contained test results of 432 FRP-wrapped plain concrete short columns with concrete strength between 18 and 55 MPa and included 194 circular, 170 square and 68 rectangular columns. This database was created to determine the parameters that affect the ultimate axial strain *ε_cu_* of the FRP jacket. For a certain confinement pressure, the lateral strain of the FRP differs for FRP jackets with different rigidity and as a result it affects the axial strain of concrete in principle [59]. The grade of the concrete is also found to affect the ultimate axial strain of concrete when all other factors are fixed [15,60]. Lower-strength concrete has a larger degree of deformability than higher-strength concrete, and thus the former displays greater confinement efficiency.

Based on these studies, the ultimate strain is affected by these parameters and can have the following mathematical form:

(1) $$\frac{\varepsilon_{cu}}{\varepsilon_{co}}\;=\;\lambda +f\left(\frac{f_{l,FRP}}{f_{co}},\frac{2r_{c}}{b},\;\frac{h}{b},\;\frac{f_{30}}{f_{co}}\right)$$

where:

$f\left(\frac{f_{l,FRP}}{f_{co}},\frac{2r_{c}}{b}\;\frac{h}{b},\frac{f_{30}}{f_{co}}\right)$ is a function of $\frac{f_{l,FRP}}{f_{co}},$ $\frac{2r_{c}}{b},$ $\frac{h}{b}$ and of $\frac{f_{30}}{f_{co}}$,

*ε_cu_* is the ultimate axial strain at the ultimate failure point,

*ε_co_* is the strain of the unconfined concrete corresponding to *f_co_*,

λ is a constant that relates the ultimate strain to the peak strain for unconfined concrete,

*f_l,FRP_* is the lateral confining pressure (MPa),

*f_co_* is the axial compressive stress of unconfined concrete (MPa),

*r_c_* is the corner radius,

*h* and *b* are the length of the longer and shorter sides of the column, respectively, and,

*f_30_* is the concrete strength of unconfined grade C30 concrete. 

All of the variables in Equation (1) are non-dimensionalized. The constant λ = 1.75 is recommended by Eurocode 2 and Ref. [2] and relates the ultimate strain to the peak strain for unconfined concrete. For the second term in Equation (1), the ultimate axial strain of concrete increases when the confinement increases, and decreases when the concrete strength increases. Additionally, when the lateral pressure provided by the FRP *f_l,FRP_* is equal to zero, or *f_co_* is equal to infinity, the confinement effectiveness is zero. So, the effects of these two terms in Equation (1) can be adequately described by two functions $\frac{f_{l,FRP}}{f_{co}},\;\frac{f_{30}}{f_{co}}$, separately. These factors mathematically are:

(2) $$\frac{\varepsilon_{cu}}{\varepsilon_{co}}\;=\;1.75+\alpha {\left(\frac{f_{l,FRP}}{f_{co}}\right)}^{\beta }{\left(\frac{f_{30}}{f_{co}}\right)}^{\gamma }f\left(\frac{2r_{c}}{b}\right)f\left(\frac{h}{b}\right)$$

in which, the values $f\left(\frac{2r_{c}}{b}\right),$ and $f\left(\frac{h}{b}\right),$ are two functions that have to be defined, while the value of *ε_co_* is calculated by the equation proposed by Popovics [61], unless the test value is available

(3) $$\varepsilon_{co}=0.000937\cdot \sqrt[{4}]{f_{co}}$$

and *α* is a coefficient equal to 12.

For square columns, substituting function $f\left(\frac{h}{b}\right)=1$ into Equation (2) yields the expression for the function $f\left(\frac{2r_{c}}{b}\right)$ as follows:

(4) $$f\left(\frac{2r_{c}}{b}\right)=\;\frac{\frac{\varepsilon_{cu}}{\varepsilon_{co}}-1.75}{12{\left(\frac{f_{l,FRP}}{f_{co}}\right)}^{0.75}{\left(\frac{f_{30}}{f_{co}}\right)}^{0.62}}$$

From the assessment of the databases by Wei and Wu [16], it is observed that the value of $\frac{2r_{c}}{b}$ increases as the corner radius ratio increases from 0 to 1, as a consequence the relationship is approximately linear. Following regression analysis using all data in the database by Wei and Wu [16], the corner radius ratio factor (function $f\left(\frac{2r_{c}}{b}\right)$) is defined as:

(5) $$f\left(\frac{2r_{c}}{b}\right)\;=\;0.36\left(\frac{2r_{c}}{b}\right)+0.64$$

Through a similar regression analysis using the full database by Wei and Wu [16], the function for the aspect ratio is obtained as

(6) $$f\left(\frac{h}{b}\right)={\left(\frac{h}{b}\right)}^{-0.3}$$

Finally, the proposed ultimate strain model for a plain square and rectangular columns is

(7) $$\frac{\varepsilon_{cu}}{\varepsilon_{co}}=1.75+12{\left(\frac{f_{l,FRP}}{f_{co}}\right)}^{0.75}{\left(\frac{f_{30}}{f_{co}}\right)}^{0.62}\left(0.36\frac{2r}{b}+0.64\right){\left(\frac{h}{b}\right)}^{-0.3}$$

and

(8) $$f_{l,FRP}=\frac{2\cdot E_{FRP}\cdot \varepsilon_{fu}\cdot t_{FRP}}{b}$$

where:

*E_FRP_* is the elastic modulus of the FRP material,

*ε_fu_* is the ultimate tensile strain by the manufacturers for the FRP material and,

*t_FRP_* is the total thickness of the jacket.

## 4. Proposed Strain Model for Concrete Columns Confined with Internal Steel Reinforcement and External FRP Jacket

### 4.1. Extended Advanced Analytical Database

The available experimental tests were carefully investigated and 11 out of them were modeled and analyzed with advanced three-dimensional finite element (3D FE) software ANSYS Explicit Dynamics software [62]. These columns were selected in order to cover the effects of a wide range of parameters, crucial for the retrofit of RC columns with FRPs for seismic resistant applications (slenderness of bars, sparse stirrups, steel quality and quantity, type and layers of FRP jacket, type of impregnation resins and reinforcing fibers, corner radius, concrete strength, predamages, etc.). The specimens were experimental tests performed by Rousakis and Karabinis [39], Ilki et al. [40] and Isleem et al. [10] and their parameters are gathered in Table 3. Then, these carefully chosen columns were modeled and analyzed for the first time pseudodynamically with three-dimensional finite elements (3D FEs). They provided numerous analytical insights into the effects of different critical parameters to allow for the enrichment of the existing databases with significant missing variables (see Table 3). More details about the analytical procedure of the 3D FE analyses are presented in Fanaradelli and Rousakis [35].

Herein, the numerous FE analytical results are further elaborated to enrich the hybrid experimental–analytical database with significant missing variables. These variables are difficult to measure in several experimental programs, as extensive local instrumentation or advanced image techniques are required, but yet are important for the prediction of ultimate strain of FRP retrofitted columns with internal steel reinforcement. The critical design parameters that were investigated through FE analyses are: (a) FRP strain at the mid-point of the side and at the level of the middle stirrup (*ε_FRP, mid, stirrup_*); (b) FRP strain at the midpoint of the side and at the midpoint level between two stirrups (*ε_FRP, mid, between_stirrups_*); (c) FRP strain at the corner of the section at the level of the middle stirrup (*ε_FRP,corner,stirrup_*); (d) FRP strain at the corner of the section at the midpoint level between two stirrups (*ε_FRP,corner, between_stirrups_*); (e) maximum strain at the stirrups (*ε_stirrup,max_*); (f) minimum strain at the longitudinal steel bars (*ε_long,min_*); (g) strain of the longitudinal bars at the mid-point level between two stirrups (*ε_long,mid_*); (h) lateral deformation (Y axis) of longitudinal steel bars at the point of stirrup; (i) lateral deformation (X axis) of longitudinal steel bars at the point of stirrup; (g) lateral deformation (Y axis) of longitudinal steel bars at the midpoint between two stirrups; (k) lateral deformation (X axis) of longitudinal steel bars at the midpoint between two stirrups and (l) lateral deformation of middle stirrup (Table 3).With these analytical results the variable deformation and strain field of the column around its perimeter at different levels may be assessed throughout loading and for different characteristic columns.

The analytical results suggested that the measured FRP tensile strains are higher at the corner of the sections at the level of the stirrups for most of the specimens (see also Table 3). Closer to the ultimate strain of the whole column is the corner FRP strain at the point between two successive stirrups. The mid-distance between two stirrups (far from end boundaries) is a critical position to place strain gauges in order to measure the representative axial strain of the steel reinforcement under axial compression in experiments (probably two or four strain gauges, symmetrically placed, are the best option if the bar buckles significantly). The axial strain of the bar at the mid distance between two stirrups is expected to be close to the average global axial strain of the column. On the other hand, the axial strains of the bars at the level of the stirrups may be far higher. The variation of the FRP strains proves that there may be in some cases local fracture of the jacket at its corner, while the global behavior of the column has not reached the ultimate failure.

The measured deformations suggest there is nonuniform bulging of the retrofitted column axially and transversely. The lateral deformation of the mid-point of the longitudinal steel bars between two stirrups in most cases is lower than half the lateral deformation at the level of the stirrups, because of the bar buckling during the axial compression. For lower FRP confinement and for lower corner radius there is a high local damage development in concrete and local bulging in both concrete and FRP. In some cases, local concrete damage may trigger the failure of the specimen prior to the fracture of the FRP jacket. It seems that the amount of the FRP confinement is an important parameter for the prediction of the ultimate strain of the RC columns. Finally, it comes out that all bars under compression exhibit and maintain strains higher than the yield one, with high variation along the axis. All stirrups under tension exceed their yield strain or are very close to it (see Fanaradelli and Rousakis [35] for more details).

**Figure 1:**
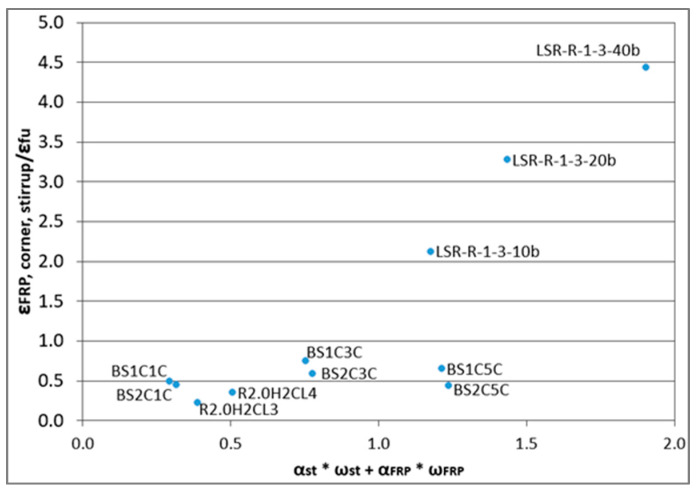
Correlation between the ratio of the FRP strain at the corner of the stirrups and the ultimate FRP strain according to the manufacturers (*ε_FRP,corner,stirrup_*/*ε_fu_*) and the sum *α_st_* × *ω_st_* + *α_FRP_* × *ω_FRP_*.

Figure 1 shows the variation of the most critical parameter as concluded by the investigation of all additional analytical findings gathered in Table 3 (variation of strains and displacements), in order to contribute to the prediction of the ultimate strain of the RC columns. The strongest correlation arose out of the ratio of the FRP strain at the corner of the section at the level of the middle stirrup to the ultimate FRP strain according to the manufacturers (*ε_FRP,corner,stirrup_*/*ε_fu_*) and the parameter *α_st_* × *ω_st_* + *α_FRP_* × *ω_FRP_*, which is the effective confinement mechanical volumetric ratio for the stirrups plus the one for the FRP. The ratio of strains arose as a combined parameter that could provide a more reliable estimate for the critical ultimate strain of the FRP at failure (significantly variable around and along the column), rather than a fictitious average FRP strain. The combined steel-FRP effective confinement mechanical volumetric ratio is chosen as a rough estimate of the different confining effects on the concrete core provided by both steel and FRP reinforcement. The rest of analytical FRP strain values at different characteristic positions revealed high scarcity and low correlation with the effective mechanical volumetric confinement ratio. Figure 1 suggests the FRP strain ratio increased with the increase of *α_st_* × *ω_st_* + *α_FRP_* × *ω_FRP_.* That is, for higher provided confinement or higher corner radius of the concrete section, etc., the effects of the internal steel reinforcement are lower. Further, as the number of the FRP layers is increased, the increase of the ratio of the strain is lower and even becomes equal to the case of low confinement. That is for a very high number of layers the quality of application affects the FRP strain variation. Characteristic is the specimens by Ilki et al. [40]. There, the analyzed columns have reached their ultimate FRP strain and the fibers have raptured in different places, leading to brittle local failure. The pseudodynamic FE analyses may provide the mechanical response of the columns beyond the original local failures and their successive behavior up to their ultimate global failure. At global failure state the columns have already reached in some positions local fracture of the FRP jacket while the analysis provides fictitious ultimate FRP strain that are higher than the ultimate strains provided by the manufacturers. While these exaggerated values are fictitious, they may reveal the general trend for this FRP strain variation, in case a phenomenological modeling approach is followed.

### 4.2. Proposed Strain Model for RC Columns Confined with FRPs

The axial strain for concrete confined with internal steel stirrups is estimated with the relationship by Mander et al. [63]

(9) $$\varepsilon_{cc,st}=\varepsilon_{co}\left[1+5\left(\frac{f_{cc,st}}{f_{co}}-1\right)\right]$$

where *f_cc,st_* is the triaxial compressive strength of concrete because of the stirrup confinement, as calculated according to the Model Code 90.

Further, the model takes into account the effects of the FRP confinement based on the Wei and Wu [16] model. The axial strain *ε_cc,st_* replaces the constant parameter λ in the Equation (1). For the prediction of axial strain at ultimate conditions for RC columns, the combined parameter *α_st,FRP_* is imported and multiplied to Equation (7) and its new form is:

(10) $$\frac{\varepsilon_{cu}}{\varepsilon_{co}}=\frac{\varepsilon_{cc,st}}{\varepsilon_{co}}+12\cdot \alpha_{st,FRP}\cdot {\left(\frac{f_{l,FRP}}{f_{co}}\right)}^{0.75}{\left(\frac{f_{30}}{f_{co}}\right)}^{0.62}\left(0.36\frac{2r_{c}}{b}+0.64\right){\left(\frac{h}{b}\right)}^{-0.3}$$

In Equation (10), *f_l,FRP_* is calculated according to Wei and Wu model and thus *ε_fu_* is the ultimate tensile strain by the manufacturer as already mentioned. Several studies [64,65,66] have shown that the ultimate FRP confining stress could be calculated using the ultimate hoop rupture strain rather than the ultimate strain of FRP coupons under direct tension. Hoop rupture strains, obtained from FRP split-disk tests and the models [25,67] based on this method are generally more accurate than those based on the FRP deformability provided by the manufacturer. However, this method is mainly used in circular specimens [64,65,66]. For rectangular cross sections, a strain efficiency factor was proposed by [7,16,19,39,68] among others, to account for the reduced average deformability of the FRP jacket. Herein, the parameter *α_st,FRP_* is proposed according to the findings discussed in Figure 1. It takes into account the number of the layers of the FRP jacket and the combined effects of steel and FRP confinement through *α_st_* × *ω_st_* + *α_FRP_* × *ω_FRP_*_._ This parameter affects the efficiency of the FRP confinement by changing indirectly the ultimate FRP strain of FRP confinement of the model by Wei and Wu [16] (*ε_fu_*). This modified ultimate FRP strain is indirectly related to the one measured at the corner of the section at the level of the stirrup (critical parameter *ε_FRP,corner,stirrup_*) of the RC columns, as resulted by the FE analyses. Therefore, in order to address the ultimate axial strain of RC concrete columns confined with FRP under cyclic loading, which is the aim of this study, *α_st,FRP_* is expressed as:

(11) $$\alpha_{st,FRP}=3.1528\cdot \frac{1}{{\left(n_{FRP}\right)}^{1.23}}\cdot \left(a_{st}\cdot \omega_{st}+a_{FRP}\cdot \omega_{\;FRP}\right)$$

where:

*α_st_* is the confinement effectiveness factor for the stirrups,

*α_FRP_* is the confinement effectiveness factor for the FRP jacket,

*ω_st_* is the mechanical volumetric ratio of the stirrups,

*ω_FRP_* is the mechanical volumetric ratio of the FRP jacket,

*n_FRP_* is the number of the layers of the FRP material.

The parameter *α_st,FRP_* ranged from 0.29 to 1.55 for the 11 specimens. Herein, it should be reminded that in some cases of the 3D FE analyses [35], the local failure of the concrete core may trigger the global failure of the column or several prior local failures of the FRP jacket were revealed. Such issues need further investigation.

**Figure 2:**
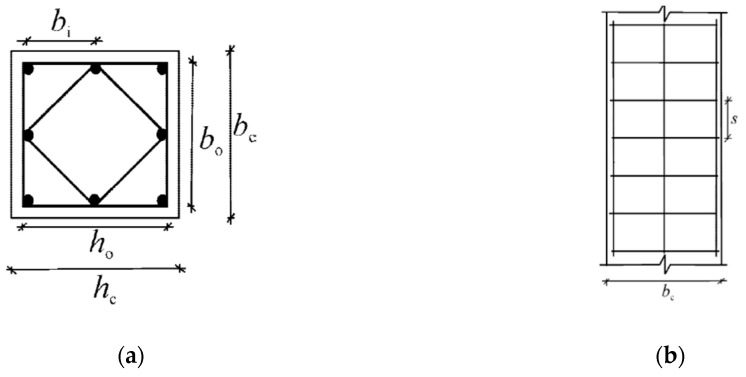
(**a**) Horizontal and (**b**) vertical cross-section of column with non-uniform distribution of confining stresses (EN 1998-1:2004 [69]).

The confinement effectiveness factor a_st_ for the stirrups is calculated according to the EN 1998-1:2004 [69]

(12) $$a_{st}=\;a_{n}*a_{s}=\left(\;1-\sum \frac{b_{i}^{2}}{6h_{0}b_{0}}\right)\left(1-\frac{1}{2}\frac{s}{b_{0}}\right)\left(1-\frac{1}{2}\frac{s}{h_{0}}\right)$$

where:

*b_i_* is the distance between consecutive bars engaged by a corner of a tie or by a cross-tie

in a column with b_i_ < 200 mm (see Figure 2a),

*b_o_* is the width of confined core (to the centerline of the hoops),

*h_o_* is the depth of confined core (to the centerline of the hoops),

*n* is the total number of tied longitudinal bars and,

*s* is the distance between two successive stirrups (see Figure 2b) with $s<\frac{b_{0}}{2}$.

The parameter *ω_w_* is the actual mechanical volumetric ratio of confining reinforcement (EN 1998-1:2004 [69])

(13) $$\omega_{w}=\frac{volume\;of\;confining\;hoops}{volume\;of\;concrete\;core}\cdot \frac{f_{y,trans}}{f_{co}}$$

where *f_y,trans_* is the stress of transverse reinforcement (yield stress of steel stirrup or stress of FRP, *E_FRP_* × *ε_fu_*).

The parameter *α_FRP_* is the confinement effectiveness coefficient according to *fib* Bulletin [70]

(14) $$\alpha_{FRP}=1-\frac{b\prime^{2}+h\prime^{2}}{3\cdot A_{g}\cdot \left(1-\rho_{sg}\right)}$$

where

*b*’ and *h*’ are the clear distances without the rounded corners (*b*’ = *b* – 2 × r_c_ and *h*’ = *h* − 2 × *r_c_*),

*A_g_* is the gross cross-sectional area and,

*ρ_sg_* is the reinforcement ratio of the longitudinal steel reinforcement with respect to the gross cross-sectional area ($\rho_{sg}=\frac{A_{s}}{A_{g}}$).

## 5. Performance of the Proposed Model

The ultimate axial strain of the specimens under cyclic axial loading is predicted using the proposed model. The performance of the model is assessed based the average absolute error (AAE) calculated with the following expression:

(15) $$AAE=\frac{\sum_{i=1}^{N}\frac{|\varepsilon_{cu,TH}-\varepsilon_{cu,EXP}|}{\varepsilon_{cu,EXP}}}{N}$$

where

*ε_cu,TH_* is the theoretical value of concrete ultimate strain,

*ε_cu,EXP_* is the corresponding experimental value and,

*N* = total number of data.

The second parameter for the average ratio (AR) is defined as:

(16) $$AR=\frac{\sum_{i=1}^{N}\frac{\varepsilon_{cu,TH}}{\varepsilon_{cu,EXP}}}{N}$$

Through the AAE, the model absolute error with respect to the experimental data was evaluated. The average ratio provides significant additional information; in fact, it shows if the predicted quantity overestimates (AR > 1) or underestimates (AR < 1) the experimental quantity. The combination of AR close to unity and AAE close to zero suggests the model is accurate enough.

**Figure 3:**
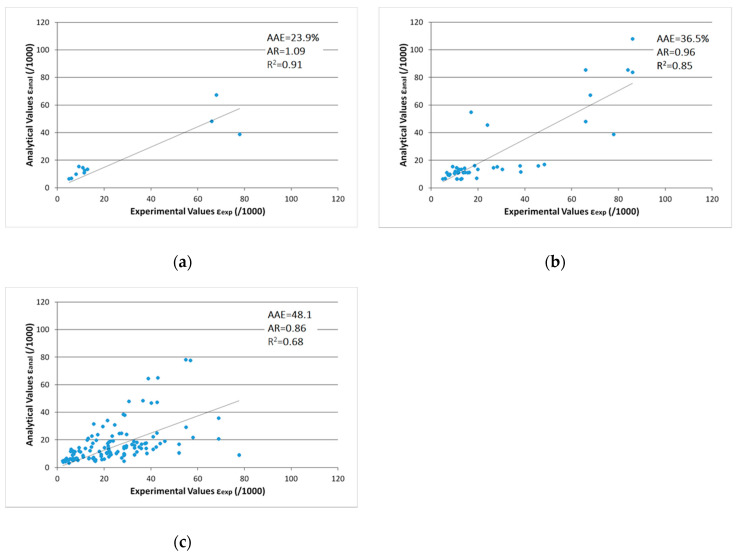
Analytical versus experimental ultimate axial strain for the proposed model (**a**) for the 11 columns analyzed with 3D FE; (**b**) for the total of 44 columns under cyclic axial loading; and (**c**) for the total of 130 columns under monotonic axial loading.

The proposed model provides the ultimate axial strain of the 11 columns being analyzed with 3D FE, with an average absolute error of 23.9% and average ratio of 1.09 (Figure 3a). The performance of the Wei and Wu [16] model prior to the proposed modifications was AAE of 61.8% and AR of 1.36, for the same specimens. In addition to that, for the total of the 44 specimens under cyclic axial loading, the AAE of the proposed model was 36.5% and the AR was 0.96 (Figure 3b), while before the incorporation of the parameter *a_st,FRP_* the AAE of the most accurate existing model (Wei and Wu [16]) was 53.4% and AR was 1.18. The regression coefficient for the 11 FE analyzed columns was 0.91 and for the 44 columns under cyclic axial loading was 0.85.

The proposed model improved the performance over the 130 RC square and rectangular concrete columns under monotonic axial compression gathered in the second database. The average values provided by the Wei and Wu [16] model before the modification were 69.7% and 1.23 for the AAE and AR respectively. The AAE of the modified model was 48.1% and the AR was 0.86 (Figure 3c). The modified model, while not oriented for columns under monotonic loading, provided better predictive performance than the models by Shehata et al. [41] (AAE = 62.1% and AR = 1.09), Chaallal et al. [42] (AAE = 69.5% and AR = 1.21), Lam and Teng [6] (AAE = 75.5% and AR=1.37), Vintzileou and Panagiotidou [18] (AAE = 76.2% and AR = 1.36), Cao et al. [47] (AAE = 68.7% and AR = 1.33) and Lim and Ozbakkaloglu [7] (AAE = 57.1% and AR = 1.07) that were used through the assessment of the database for the prediction of the ultimate strain for columns under monotonic axial loading [28]. The regression coefficient for the 130 specimens under monotonic axial compression was 0.68.

Further, the proposed ultimate strain model provided satisfactory predictions for 27 of the RC columns of the database with descending second branches under cyclic axial loading, revealing an AAE equal to 31.0% and AR 0.93. This performance was very important as these columns constitute the case commonly met in real practice of FRP confined RC columns of a non-circular section with a low achievable corner radius of the section and inadequate steel reinforcement detailing in seismic resistant redesign. For the 97 of columns with descending second branches under monotonic axial loading the AAE was 54.1% and AR was 1.06. It seems that the proposed model provides the best predictive performance for columns with descending second branches under cyclic axial load.

## 6. Conclusions

The present study investigated the effects of the internal steel reinforcement and of the external FRP confinement on the ultimate axial strain of RC columns of the square and rectangular section, subjected to cyclic axial loading. The assessed strain models in the previous work by Fanaradelli et al. [28,29] are proved to be inefficient and presented AAEs higher than 50%. The model by Wei and Wu [16], which can be used for columns with ascending and descending second stress–strain branches, had the best overall predictive performance among all investigated models and herein was used as the basis for suitable modifications. Fanaradelli and Rousakis [35] conducted advanced 3D FE analyses of 11 characteristic RC columns confined with FRP and investigated the critical parameters important for the improvement of strain predictions. These columns cover a wide range of different characteristics, which are important for the retrofit of RC columns with FRPs for seismic resistant applications. These parameters are the internal steel rebar of different slenderness, volumetric ratio (and quality) under compression, the stirrups placed at different spacing, the external FRP jacket applied at different layers and the concrete core with different side ratios, corner radii and concrete strength on the ultimate axial strain of the columns.

The results of the 3D FE analyses allowed the enrichment of the existing database (forming a hybrid experimental–analytical one) for columns under cyclic compression with significant additional parameters. These parameters are the strain of the FRP materials at characteristic levels and positions around the specimens (related to the position of stirrups), and the lateral deformations and strains that are developed on the steel bars and stirrups at different characteristic positions. The strongest correlation comes from the ratio of the FRP strain at the corner of the section at the level of the middle stirrup to the ultimate FRP strain according to the manufacturers (*ε_FRP,corner,stirrup_*/*ε_fu_*) and the parameter *α_st_* × *ω_st_* + *α_FRP_* × *ω_FRP_*, which is the effective confinement mechanical volumetric ratio for the stirrups plus the one for the FRP. Based on this, the parameter *α_st,FRP_* was proposed, which also includes the number of the layers of the FRP material.

The proposed model herein is a modification of the model by Wei and Wu [16] (originally proposed for plain concrete columns). The new model utilizes the Mander et al. [63] model for the effects of the steel stirrup confinement and includes a new parameter α_st,FRP_, which affects the efficiency of the FRP confinement by changing indirectly the critical FRP strain of FRP confinement at ultimate of the model by Wei and Wu [16], based on the FE analytical results, in order to address the ultimate axial strain of RC concrete columns under cyclic loading. The achieved enhancement of the performance of the proposed model may contribute to safe seismic resistant redesign as these columns constitute the case commonly met in the real practice of FRP confined RC columns of the non-circular section with a low achievable corner radius of the section and inadequate steel reinforcement detailing in seismic resistant redesign. The modified model predicts the ultimate strain values for the 11 columns of the 3D FE investigation with an average absolute error of 23.9% and average ratio of 1.09. For all the columns of the database under cyclic axial loading the corresponding predictions provided AAE of 36.5% and AR of 0.96. Finally, for columns with descending second branches the model seemed to be more accurate with AAE being 31.0% and AR being 0.93. This performance is very important as these columns constitute the case commonly met in real practice of FRP confined RC columns of the non-circular section with a low achievable corner radius of the section and inadequate steel reinforcement detailing in seismic resistant redesign. The proposed model provides improved predictions for 130 RC square and rectangular concrete columns under monotonic axial compression but the AAE remained as high as around 50%. Future research should identify additional critical parameters and suitably address them experimentally and analytically to improve the ultimate strain predictions.

## Figures and Tables

**Table 1:** Geometrical and mechanical data of 44 reinforced concrete (RC) square and rectangular columns under cyclic axial loading.

Reference	Specimen	h (mm)	b (mm)	H (mm)	*r_c_* (mm)	*t_FRP_* (mm)	*E_FRP_* (GPa)	Long.	*f_y, long_* (MPa)	Stirrups	*f_y, stirrup_* (MPa)	*f_co_* (MPa)	*f_cc_* (MPa)	*ε_cc_* (‰)	*f_cu_* (MPa)	*ε_cu_* (‰)	*ε_cu, pred._*^1^ (‰)	AAE (%)	AR
Wang et al. [37]	S1H1L2C	305	305	915	30	0.334	240	12Φ12	340	Φ6/80	397	25.50	33.90	3.87	27.90	10.00	10.50	5.0	1.05
	S1H1L3C	305	305	915	30	0.501	240	12Φ12	340	Φ6/80	397	25.50	36.50	4.87	33.90	38.30	11.53	69.9	0.30
	S2H1L1P	204	204	612	20	0.167	240	8Φ10	312	Φ6/120	397	25.50	33.10	4.85	31.00	19.90	13.42	32.6	0.67
	S2H1L1C	204	204	612	20	0.167	240	8Φ10	312	Φ6/120	397	25.50	33.50	5.35	32.80	30.43	13.42	55.9	0.44
	S2H1L2P	204	204	612	20	0.334	240	8Φ10	312	Φ6/120	397	25.50	43.80	38.01	43.80	38.01	15.96	58.0	0.42
	S2H1L2C	204	204	612	20	0.334	240	8Φ10	312	Φ6/120	397	25.50	38.60	45.74	38.60	45.74	15.96	65.1	0.35
	S2H2L1C	204	204	612	20	0.167	240	8Φ10	312	Φ6/60	397	25.50	34.80	26.61	34.80	26.61	14.57	45.3	0.55
	S2H2L2C	204	204	612	20	0.334	240	8Φ10	312	Φ6/60	397	25.50	41.40	48.39	41.40	48.39	16.95	65.0	0.35
Rousakis et al. [38]	C16_S8_S5,5_75_L2	250	150	750	25	0.344	76	6Φ8	220	Φ5.5/75	220	24.40	30.16	6.30	25.64	7.73	9.31	20.4	1.20
	C16_S8_S5,5_150_L2	250	150	750	25	0.344	76	6Φ8	220	Φ5.5/150	220	24.40	28.94	5.40	24.60	7.82	9.09	16.3	1.16
	C16_B8_S5,5_75_L2	250	150	750	25	0.344	76	6Φ8	500	Φ5.5/75	220	24.40	30.56	5.37	25.98	7.28	9.31	27.9	1.28
	C16_S8_S5,5_75_L4	250	150	750	25	0.688	76	6Φ8	220	Φ5.5/75	220	24.40	32.95	7.00	28.01	16.20	11.16	31.1	0.69
	C16_S8_S5,5_150_L4	250	150	750	25	0.688	76	6Φ8	220	Φ5.5/150	220	24.40	31.04	7.00	26.38	15.66	10.96	30.0	0.70
	C16_B8_S5,5_75_L4	250	150	750	25	0.688	76	6Φ8	500	Φ5.5/75	220	24.40	31.00	7.95	26.35	14.60	11.16	23.6	0.76
	C16_B8_S5,5_150_L4#1	250	150	750	25	0.688	76	6Φ8	500	Φ5.5/150	220	24.40	32.00	5.75	27.20	13.76	10.96	20.3	0.80
	C16_B8_S5,5_150_L4#2	250	150	750	25	0.688	76	6Φ8	500	Φ5.5/150	220	24.40	32.64	6.24	27.74	11.67	10.96	6.1	0.94
Isleem et al. [10]	R1.5H2CL2	300	200	1000	30	0.334	240	6Φ16	360	Φ8/90	373	46.30	52.90	2.95	45.50	11.10	6.49	41.5	0.58
	R1.5H2CL3	300	200	1000	30	0.501	240	6Φ16	360	Φ8/90	373	46.30	57.20	3.83	57.30	19.44	6.92	64.4	0.36
	R1.5H1CL2	300	200	1000	30	0.334	240	6Φ16	360	Φ8/180	373	46.30	54.10	3.18	46.60	12.70	6.27	50.7	0.49
	R1.5H1CL3	300	200	1000	30	0.501	240	6Φ16	360	Φ8/180	373	46.30	54.70	3.53	54.30	13.09	6.70	48.8	0.51
	R2.0H2CL3	400	200	1000	40	0.501	240	8Φ16	360	Φ8/100	373	46.30	54.40	4.03	39.36	5.00	6.45	28.9	1.29
	R2.0H2CL4	400	200	1000	40	0.668	240	8Φ16	360	Φ8/100	373	46.30	57.80	3.93	39.36	6.00	6.73	12.2	1.12
	R2.0H1CL4	400	200	1000	40	0.668	240	8Φ16	360	Φ8/200	373	46.30	53.90	3.21	39.36	11.00	6.39	41.9	0.58
Rousakis and Karabinis [39]	BS1C1C	200	200	320	30	0.117	240	4Φ14	500	Φ8/200	500	25.50	37.34	2.38	31.74	7.95	9.82	23.5	1.24
	BS1C3C	200	200	320	30	0.351	240	4Φ14	500	Φ8/200	500	25.50	40.96	6.80	34.85	11.63	12.51	7.6	1.08
	BS1C5C	200	200	320	30	0.585	240	4Φ14	500	Φ8/200	500	25.50	55.35	10.13	53.60	10.83	14.57	34.5	1.35
	BS1G3C	200	200	320	30	0.462	73	4Φ14	500	Φ8/200	500	25.50	35.41	6.17	33.92	6.71	11.03	64.4	1.64
	BS1G6C	200	200	320	30	0.924	73	4Φ14	500	Φ8/200	500	25.50	57.73	8.33	46.86	11.86	13.42	13.1	1.13
	BS1G9C	200	200	320	30	1.386	73	4Φ14	500	Φ8/200	500	25.50	58.34	10.04	49.98	28.22	15.31	45.7	0.54
	BS2C1C	200	200	320	30	0.117	240	4Φ14	500	Φ8/95	500	25.50	42.99	3.29	39.37	11.44	10.87	5.0	0.95
	BS2C3C	200	200	320	30	0.351	240	4Φ14	500	Φ8/95	500	25.50	50.68	12.90	50.68	12.90	13.40	3.9	1.04
	BS2C5C	200	200	320	30	0.585	240	4Φ14	500	Φ8/95	500	25.50	59.29	9.17	59.21	9.17	15.40	68.0	1.68
	BS2G3C	200	200	320	30	0.462	73	4Φ14	500	Φ8/95	500	25.50	50.10	10.28	50.10	10.28	11.94	16.2	1.16
	BS2G6C	200	200	320	30	0.924	73	4Φ14	500	Φ8/95	500	25.50	66.82	14.28	66.85	14.28	14.25	0.2	1.00
	BS2G9C	200	200	320	30	1.386	73	4Φ14	500	Φ8/95	500	25.50	75.17	18.03	72.87	18.52	16.12	13.0	0.87
Ilki et al. [40]	LSR-R-1-1-40b	250	250	500	40	0.165	230	4Φ14	345	Φ8/200	476	10.83	20.39	24.00	20.39	24.00	45.55	89.8	1.90
	LSR-R-1-3-10b	250	250	500	10	0.495	230	4Φ14	345	Φ8/200	476	10.83	26.60	78.00	26.60	78.00	38.71	50.4	0.50
	LSR-R-1-3-20b	250	250	500	20	0.495	230	4Φ14	345	Φ8/200	476	10.83	31.12	66.00	31.12	66.00	48.11	27.1	0.73
	LSR-R-1-3-40b	250	250	500	40	0.495	230	4Φ14	345	Φ8/200	476	10.83	41.17	68.00	41.17	68.00	67.21	1.2	0.99
	LSR-R-1-5-40b	250	250	500	40	0.825	230	4Φ14	345	Φ8/200	476	10.83	48.07	86.00	48.07	86.00	83.71	2.7	0.97
	LSR-R-2-1-40b	300	150	500	40	0.165	230	4Φ12	339	Φ8/175	476	11.16	11.74	17.00	11.74	17.00	54.81	222.4	3.22
	LSR-R-2-3-40b-PD	300	150	500	40	0.495	230	4Φ12	339	Φ8/175	476	11.16	40.48	84.00	40.48	84.00	85.37	1.6	1.02
	LSR-R-2-3-40b	300	150	500	40	0.495	230	4Φ12	339	Φ8/175	476	11.16	26.80	66.00	26.80	66.00	85.37	29.4	1.29
	LSR-R-2-5-40b	300	150	500	40	0.825	230	4Φ12	339	Φ8/175	476	11.16	36.25	86.00	36.25	86.00	107.88	25.5	1.25

^1^*ε_cu,pred._* is the ultimate axial strain of the proposed model.

**Table 2:** Geometrical and mechanical data of 130 RC square and rectangular columns under monotonic axial loading.

Reference	Specimen	h (mm)	b (mm)	H (mm)	*r_c_* (mm)	*t_FRP_* (mm)	*E_FRP_* (GPa)	Long.	*f_y, long_* (MPa)	Stirrups	*f_y, stirrup_* (MPa)	*f_co_* (MPa)	*f_cc_* (MPa)	*ε_cc_* (‰)	*f_cu_* (MPa)	*ε_cu_* (‰)	*ε_cu, pred._* (‰)	AAE (%)	AR
Tastani et al. [46]	MRC2.1	200	200	320	25	0.26	235	4Φ12	562	Φ6/75	220	20.50	36.28	7.52	30.84	7.52	11.28	50.1	1.50
	MRC2.2	200	200	320	25	0.26	235	4Φ12	562	Φ6/75	220	20.50	37.74	6.56	32.08	6.56	11.81	80.0	1.80
	MRC2.3	200	200	320	25	0.26	235	4Φ12	562	Φ6/75	220	20.50	38.03	6.11	32.33	6.11	11.98	96.1	1.96
	FRC2.1	200	200	320	25	0.26	235	4Φ12	562	Φ6/140	220	22.20	33.73	4.90	28.67	7.00	9.72	38.8	1.39
	FRC2.2	200	200	320	25	0.26	235	4Φ12	562	Φ6/140	220	22.20	38.50	4.90	32.73	7.00	11.51	64.5	1.64
	FRC2.3	200	200	320	25	0.26	235	4Φ12	562	Φ6/140	220	22.20	35.02	4.90	29.77	7.00	10.18	45.4	1.45
	FSC2.1	200	200	320	25	0.26	235	4Φ12	562	Φ6/140	220	22.20	30.68	11.00	26.08	11.00	8.61	21.7	0.78
	FSC2.2	200	200	320	25	0.26	235	4Φ12	562	Φ6/140	220	22.20	31.88	16.80	27.09	28.56	8.97	68.6	0.31
	FSC2.3	200	200	320	25	0.26	235	4Φ12	562	Φ6/140	220	22.20	33.59	16.80	28.55	28.56	9.64	66.2	0.34
	FSC4.1	200	200	320	25	0.52	235	4Φ12	562	Φ6/140	220	22.20	37.02	11.30	31.47	11.98	13.64	13.9	1.14
	FSC4.2	200	200	320	25	0.52	235	4Φ12	562	Φ6/140	220	22.20	39.75	28.50	33.79	28.50	14.88	47.8	0.52
	FSC4.3	200	200	320	25	0.52	235	4Φ12	562	Φ6/140	220	22.20	37.26	28.50	31.68	28.50	13.74	51.8	0.48
	FSC2.1	200	200	320	25	0.26	235	4Φ12	562	Φ6/140	220	22.20	32.47	28.50	27.60	28.50	9.19	67.7	0.32
	FSC2.2	200	200	320	25	0.26	235	4Φ12	562	Φ6/140	220	22.20	34.17	28.50	29.04	28.50	9.87	65.4	0.35
	FSC2.3	200	200	320	25	0.26	235	4Φ12	562	Φ6/140	220	22.20	31.32	28.50	26.62	28.50	8.83	69.0	0.31
	FSG2.1	200	200	320	25	0.34	75	4Φ12	562	Φ6/140	220	22.20	29.93	28.50	25.44	28.50	4.64	83.7	0.16
	FSG2.2	200	200	320	25	0.34	75	4Φ12	562	Φ6/140	220	22.20	29.56	11.00	25.12	16.28	4.54	72.1	0.28
	FSG2.3	200	200	320	25	0.34	75	4Φ12	562	Φ6/140	220	22.20	31.30	14.10	26.60	15.9	4.97	68.8	0.31
	FSG4.1	200	200	320	25	0.68	75	4Φ12	562	Φ6/140	220	22.20	37.93	10.10	32.24	11.11	7.70	30.7	0.69
	FSG4.2	200	200	320	25	0.68	75	4Φ12	562	Φ6/140	220	22.20	32.66	15.50	27.76	20.00	6.20	69.0	0.31
	FSG4.3	200	200	320	25	0.68	75	4Φ12	562	Φ6/140	220	22.20	34.58	8.04	29.39	8.04	6.76	15.9	0.84
	FRG2.1	200	200	320	25	0.34	75	4Φ12	562	Φ6/140	220	22.20	34.29	12.80	29.15	16.38	5.66	65.5	0.35
	FRG2.2	200	200	320	25	0.34	75	4Φ12	562	Φ6/140	220	22.20	34.58	15.60	29.39	19.03	5.76	69.7	0.30
	FRG2.3	200	200	320	25	0.34	75	4Φ12	562	Φ6/140	220	22.20	32.79	4.29	27.87	6.52	5.31	18.6	0.81
Esfahani and Kianoush [47]	SA	180	180	850	0	2.000	72	6Φ10	541	Φ10/160	365	35.00	43.74	8.70	37.18	77.80	8.98	88.5	0.12
	SB	180	180	850	0	2.000	72	6Φ10	541	Φ10/160	365	35.00	41.45	3.88	35.23	6.50	8.98	38.1	1.38
	SC	180	180	850	12	2.000	72	6Φ10	541	Φ10/160	365	35.00	50.20	12.82	42.67	14.00	12.07	13.8	0.86
Pessiki et al. [48]	TS2	457	457	1830	38	2.16	19	8Φ22	400	Φ9.5/366	400	31.38	36.40	3.00	30.94	2.52	3.99	58.4	1.58
	TS3	457	457	1830	38	2.799	22	8Φ22	400	Φ9.5/366	400	31.42	35.50	2.50	30.18	3.51	4.21	20.0	1.20
	TS4	457	457	1830	38	3.045	38	8Φ22	400	Φ9.5/366	400	32.24	37.40	2.10	31.79	2.26	4.83	113.6	2.14
Tan [49]	M02CP	420	115	1500	30	0.33	228	8Φ13	495	Φ6/100	365	23.3	39.05	3.15	33.93	3.15	5.47	73.7	1.74
	M12G	420	115	1500	30	0.706	72	8Φ13	495	Φ6/100	365	25.2	35.01	3.08	34.03	3.08	5.02	63.0	1.63
	M22G	420	115	1500	30	0.706	72	8Φ13	495	Φ6/100	365	18.8	38.74	4.17	35.30	4.17	5.97	43.2	1.43
	M13G	420	115	1500	30	1.059	72	8Φ13	495	Φ6/100	365	24.4	32.93	4.66	31.37	4.66	5.28	13.2	1.13
	M23G	420	115	1500	30	1.059	72	8Φ13	495	Φ6/100	365	24	37.52	6.74	35.35	6.74	5.33	20.9	0.79
	M22GP	420	115	1500	30	0.706	72	8Φ13	495	Φ6/100	365	23.7	35.81	2.89	30.75	2.89	5.17	79.1	1.79
	M22GPA	420	115	1500	30	0.706	72	8Φ13	495	Φ6/100	365	24.4	37.62	3.29	35.98	3.29	5.09	54.7	1.55
	M23GP	420	115	1500	30	1.059	72	8Φ13	495	Φ6/100	365	24.4	39.32	8.75	34.99	8.75	5.28	39.6	0.60
Cole and Belarbi [50]	2GFRP-Rect1/1-Ti178mm-ρ1.6	181	181	914	13	0.706	72	4Φ12.7	400	Φ6.4/78	427	21.00	39.11	31.86	39.05	31.86	16.60	47.9	0.52
	3GFRP-Rect1/1-Ti178mm-ρ1.6	181	181	914	13	1.059	72	4Φ12.7	400	Φ6.4/78	427	21.00	46.75	32.70	46.69	32.70	18.72	42.7	0.57
	1AFRP-Rect1/1-Ti178mm-ρ1.6	181	181	914	13	0.279	117	4Φ12.7	400	Φ6.4/78	427	21.00	43.39	7.71	38.37	14.55	14.77	1.5	1.02
	2AFRP-Rect1/1-Ti178mm-ρ1.6	181	181	914	13	0.558	117	4Φ12.7	400	Φ6.4/78	427	21.00	46.22	15.17	46.16	15.17	17.38	14.6	1.15
	3AFRP-Rect1/1-Ti178mm-ρ1.6	181	181	914	13	0.837	117	4Φ12.7	400	Φ6.4/78	427	21.00	54.36	16.60	54.30	16.60	19.65	18.3	1.18
de Diego et al. [51]	2GW(13)	150	150	600	25	0.16	70	4Φ6	400	Φ6/100	400	13	21.00	37.50	21.00	37.50	17.24	54.0	0.46
	2GM(13)	150	150	600	25	0.16	70	4Φ6	400	Φ6/100	400	13	27.98	21.50	27.98	21.50	17.24	19.8	0.80
	3GP(16,5)	150	150	600	25	0.16	70	4Φ6	400	Φ6/100	400	16.5	29.32	5.70	29.32	5.70	11.58	103.1	2.03
	4GM(16,5)	150	150	600	25	0.16	70	4Φ6	400	Φ6/100	400	16.5	26.08	17.90	26.08	17.90	11.58	35.3	0.65
	5GW(16,7)	150	150	600	25	0.16	70	4Φ6	400	Φ6/100	400	16.7	26.96	22.30	26.96	22.30	11.37	49.0	0.51
	5CM(16,7)	150	150	600	25	0.24	242	4Φ6	400	Φ6/100	400	16.7	32.23	21.40	32.23	21.40	34.03	59.0	1.59
	6GW(17,5)	150	150	600	25	0.16	70	4Φ6	400	Φ6/100	400	17.5	27.02	21.30	27.02	21.30	10.60	50.2	0.50
	6GP(17,5)	150	150	600	25	0.16	70	4Φ6	400	Φ6/100	400	17.5	32.24	22.60	32.24	22.60	10.60	53.1	0.47
Wang and Hsu [52]	CS2	300	300	900	30	2.54	21	4Φ20	439	Φ10/180	365	18.90	28.30	15.85	24.05	22.62	18.99	16.1	0.84
	CS6	300	300	900	30	7.62	21	4Φ20	439	Φ10/180	365	18.90	45.11	42.60	45.11	42.60	24.86	41.6	0.58
	CR2	450	300	900	30	2.54	21	6Φ20	439	Φ10/180	365	18.90	26.81	4.88	22.78	21.77	15.42	29.2	0.71
	CR6	450	300	900	30	7.62	21	6Φ20	439	Φ10/180	365	18.90	33.48	23.86	33.48	23.86	19.05	20.2	0.80
Kumutha et al. [53]	2S1	160	160	750	0	2.2	11	4Φ10	478	Φ6/125	250	27.45	51.60	3.91	51.60	3.91	6.55	67.5	1.67
	2R1.25	200	160	750	0	2.2	11	4Φ10	478	Φ6/125	250	27.45	50.11	8.40	50.11	8.40	6.64	21.0	0.79
	2R1.66	265.6	160	750	0	2.2	11	4Φ10	478	Φ6/125	250	27.45	46.40	5.00	46.40	5.00	3.31	33.9	0.66
Ilki et al. [54]	LS-R-1-200-3-40	250	250	500	40	0.495	230	4Φ14	345	Φ8/200	476	15.9	37.40	55.00	37.40	55.00	29.14	47.0	0.53
	LS-R-1-200-5-40	250	250	500	40	0.825	230	4Φ14	345	Φ8/200	476	15.9	51.60	69.00	51.60	69.00	35.64	48.3	0.52
	NS-R-1-200-3-40	250	250	500	40	0.495	230	4Φ14	345	Φ8/200	476	31	42.00	33.00	42.00	33.00	9.23	72.0	0.28
	NS-R-1-200-5-40	250	250	500	40	0.825	230	4Φ14	345	Φ8/200	476	31	58.30	52.00	58.30	52.00	10.57	79.7	0.20
	NS-R-2-175-3-40	300	150	500	40	0.495	230	4Φ14	339	Φ8/145	476	31	40.60	34.00	40.60	34.00	11.11	67.3	0.33
	NS-R-2-175-5-40	300	150	500	40	0.825	230	4Φ14	339	Φ8/145	476	31	47.80	41.00	47.80	41.00	13.07	68.1	0.32
Faustino et al. [55]	QR1C1	150	150	750	0	0.352	217	8Φ6	587	Φ3/100	500	25.98	33.78	6.86	33.11	29.40	14.42	50.9	0.49
	QR1C2	150	150	750	0	0.352	217	8Φ6	587	Φ3/100	500	27.81	33.38	6.84	31.69	21.93	12.98	40.8	0.59
	QR1C3	150	150	750	0	0.352	217	8Φ6	587	Φ3/100	500	26.91	34.98	3.64	32.98	21.85	13.75	37.1	0.63
	QR2C1	150	150	750	20	0.352	217	8Φ6	587	Φ3/100	500	26.84	51.00	26.49	51.00	26.49	24.50	7.5	0.92
	QR2C2	150	150	750	20	0.352	217	8Φ6	587	Φ3/100	500	27.23	57.18	29.58	57.18	29.58	23.80	19.5	0.80
	QR2C3	150	150	750	20	0.352	217	8Φ6	587	Φ3/100	500	26.72	53.44	27.42	53.44	27.42	24.75	9.7	0.90
	QR3C1	150	150	750	38	0.352	217	8Φ6	587	Φ3/100	500	28.22	64.91	15.53	64.91	15.53	31.39	102.1	2.02
	QR3C2	150	150	750	38	0.352	217	8Φ6	587	Φ3/100	500	28.50	68.39	24.48	68.39	24.48	30.71	25.5	1.25
	QR3C3	150	150	750	38	0.352	217	8Φ6	587	Φ3/100	500	28.96	66.60	19.44	66.60	19.44	29.58	52.2	1.52
Wang et al. [37]	S1H1L2M	305	305	915	18	0.334	240	12Φ12	340	Φ6/80	397	25.5	34.90	4.34	30.70	22.69	9.37	58.7	0.41
	S1H1L3M	305	305	915	18	0.501	240	12Φ12	340	Φ6/80	397	25.5	36.90	4.28	33.30	25.21	10.20	59.5	0.40
	S1H2L2M	305	305	915	18	0.334	240	12Φ12	340	Φ6/40	397	25.5	35.50	4.16	30.90	21.04	10.56	49.8	0.50
	S1H2L3M	305	305	915	18	0.501	240	12Φ12	340	Φ6/40	397	25.5	37.20	5.29	35.80	25.84	11.32	56.2	0.44
	S1H1L2D1	305	305	915	18	0.334	240	12Φ12	340	Φ6/80	397	25.5	34.70	4.52	30.10	18.72	9.37	50.0	0.50
	S1H1L2D2	305	305	915	18	0.334	240	12Φ12	340	Φ6/80	397	25.5	35.50	4.32	30.30	22.96	9.37	59.2	0.41
	S1H1L3D1	305	305	915	18	0.501	240	12Φ12	340	Φ6/80	397	25.5	36.50	4.49	32.80	21.22	10.20	51.9	0.48
	S1H1L3D2	305	305	915	18	0.501	240	12Φ12	340	Φ6/80	397	25.5	34.70	4.56	33.90	38.3	10.20	73.4	0.27
	S2H1L2M	204	204	612	12	0.334	240	8Φ10	312	Φ6/120	397	25.5	40.00	35.88	40.00	35.88	13.71	61.8	0.38
	S2H1L2P	204	204	612	12	0.334	240	8Φ10	312	Φ6/120	397	25.5	43.80	38.01	43.80	38.01	13.71	63.9	0.36
	S2H2L2M	204	204	612	12	0.334	240	8Φ10	312	Φ6/60	397	25.5	40.80	42.30	40.80	42.30	14.68	65.3	0.35
Bournas et al. [56]	s200_R2	200	200	380	25	0.34	225	4Φ12	563	Φ8/200	563	22.13	37.27	12.80	37.27	12.80	19.79	54.6	1.55
	s200_R3	200	200	380	25	0.51	225	4Φ12	563	Φ8/200	563	22.13	44.65	14.80	44.65	14.80	22.71	53.5	1.53
	s100_R2	200	200	380	25	0.34	225	4Φ12	563	Φ8/100	563	22.13	41.97	13.20	41.97	13.20	20.91	58.4	1.58
	s100_R3	200	200	380	25	0.51	225	4Φ12	563	Φ8/100	563	22.13	45.23	17.20	45.23	17.20	23.75	38.1	1.38
Ilki et al. [40]	NSR-R-1-050-3-40	250	250	500	40	0.495	230	4Φ14	345	Φ8/50	476	23.44	44.67	33.00	44.67	33.00	15.90	51.8	0.48
	NSR-R-1-100-3-40	250	250	500	40	0.495	230	4Φ14	345	Φ8/100	476	23.44	45.80	35.00	45.80	35.00	14.75	57.8	0.42
	NSR-R-1-200-3-40	250	250	500	40	0.495	230	4Φ14	345	Φ8/200	476	23.44	42.08	33.00	42.08	33.00	14.15	57.1	0.43
	NSR-R-1-050-5-40	250	250	500	40	0.825	230	4Φ14	345	Φ8/50	476	23.44	57.40	46.00	57.40	46.00	18.89	58.9	0.41
	NSR-R-1-100-5-40	250	250	500	40	0.825	230	4Φ14	345	Φ8/100	476	23.44	57.73	44.00	57.73	44.00	17.22	60.9	0.39
	NSR-R-1-200-5-40	250	250	500	40	0.825	230	4Φ14	345	Φ8/200	476	23.44	58.82	52.00	58.82	52.00	16.77	67.7	0.32
	NSR-R-2-050-3-40	300	150	500	40	0.495	230	4Φ12	339	Φ8/50	476	23.44	40.34	36.00	40.34	36.00	16.85	53.2	0.47
	NSR-R-2-100-3-40	300	150	500	40	0.495	230	4Φ12	339	Φ8/100	476	23.44	43.04	38.00	43.04	38.00	17.55	53.8	0.46
	NSR-R-2-175-3-40	300	150	500	40	0.495	230	4Φ12	339	Φ8/175	476	23.44	40.80	34.00	40.80	34.00	18.13	46.7	0.53
	NSR-R-2-050-5-40	300	150	500	40	0.825	230	4Φ12	339	Φ8/50	476	23.44	60.33	69.00	60.33	69.00	20.72	70.0	0.30
	NSR-R-2-100-5-40	300	150	500	40	0.825	230	4Φ12	339	Φ8/100	476	23.44	53.13	58.00	53.13	58.00	21.70	62.6	0.37
	NSR-R-2-175-5-40-A	300	150	500	40	0.825	230	4Φ12	339	Φ8/175	476	23.44	48.02	41.00	48.02	41.00	22.20	45.9	0.54
Wang et al. [57]	R200L2	200	200	600	23	0.334	240	8Φ10	356	Φ6/160	338	24.60	42.07	22.00	42.07	22.00	18.25	17.1	0.83
	R300L3	300	300	900	42	0.501	240	8Φ14	380	Φ6/105	338	22.70	39.73	29.54	39.73	29.54	15.57	47.3	0.53
	R400L4	400	400	1200	52	0.668	240	8Φ20	328	Φ6/140	338	21.10	23.84	5.91	23.84	5.91	12.97	119.5	2.19
	R300L2	300	300	900	45	0.334	240	8Φ14	380	Φ6/105	338	22.70	34.05	20.12	34.05	20.12	14.22	29.3	0.71
	R350L2	350	350	1050	48	0.334	240	8Φ16	350	Φ6/160	338	21.90	26.06	9.24	26.06	9.24	11.92	29.0	1.29
	R175L2	175	175	525	24	0.334	240	8Φ8	418	Φ6/180	338	25.20	55.69	23.43	55.69	23.43	22.62	3.4	0.97
	R350L4	350	350	1050	45	0.668	240	8Φ16	350	Φ6/160	338	21.90	32.63	9.23	32.63	9.23	14.24	54.3	1.54
Triantafillou et al. [58]	II3	450	150	800	20	2	94	6Φ12	570	Φ8/150	570	18	22.94	3.10	21.47	9.90	11.17	12.8	1.13
Eid and Raultre [9]	C30S100N2	150	150	300	15	0.762	65	4Φ8	513	Φ6/100	258	33.70	39.90	13.60	39.90	13.6	6.83	49.7	0.50
	C30S100N4	150	150	300	15	1.524	65	4Φ8	513	Φ6/100	258	33.70	57.10	22.00	57.10	22.00	7.79	64.6	0.35
	C30S50N2	150	150	300	15	0.762	65	4Φ8	513	Φ6/50	258	33.70	44.80	15.50	44.80	15.50	7.23	53.4	0.47
	C30S50N4	150	150	300	15	1.524	65	4Φ8	513	Φ6/50	258	33.70	57.50	18.70	57.50	18.70	8.15	56.4	0.44
Isleem et al. [10]	R1.5H2ML2	300	200	1000	30	0.334	240	6Φ16	360	Φ8/90	373	46.30	53.10	3.40	46.90	13.44	6.49	51.7	0.48
	R1.5H2ML3	300	200	1000	30	0.501	240	6Φ16	360	Φ8/90	373	46.30	55.30	43.00	55.40	27.47	6.92	74.8	0.25
	R1.5H1ML2	300	200	1000	30	0.334	240	6Φ16	360	Φ8/180	373	46.30	52.20	2.68	44.90	7.71	6.27	18.7	0.81
	R1.5H1ML3	300	200	1000	30	0.501	240	6Φ16	360	Φ8/180	373	46.30	54.30	3.09	54.20	15.20	6.70	55.9	0.44
	R2.0H2ML3	400	200	1000	40	0.501	240	6Φ16	360	Φ8/100	373	46.30	55.40	3.36	47.09	5.45	6.24	14.4	1.14
	R2.0H2ML4	400	200	1000	40	0.668	240	6Φ16	360	Φ8/100	373	46.30	56.90	4.40	48.37	6.98	6.53	6.5	0.93
	R2.0H1ML3	400	200	1000	40	0.501	240	6Φ16	360	Φ8/200	373	46.30	53.70	3.58	45.65	5.60	6.06	8.2	1.08
	R2.0H1ML4	400	200	1000	40	0.668	240	6Φ16	360	Φ8/200	373	46.30	53.60	3.69	45.56	6.26	6.36	1.5	1.02
Rousakis et al. [13]	AS1C3(2)	200	200	320	30	0.351	240	4Φ14	500	Φ6/200	220	13.40	34.72	28.76	34.72	28.76	37.83	31.5	1.32
	AS1C5(2)	200	200	320	30	0.585	240	4Φ14	500	Φ6/200	220	13.40	45.84	40.21	45.84	40.21	46.73	16.2	1.16
	AS1G3(2)	200	200	320	30	0.4614	73	4Φ14	500	Φ6/200	220	13.40	30.09	30.60	30.09	30.60	47.90	56.5	1.57
	AS1G6(2)	200	200	320	30	0.9228	73	4Φ14	500	Φ6/200	220	13.40	44.12	38.92	44.12	38.92	64.47	65.7	1.66
	AS1G9(2)	200	200	320	30	1.3842	73	4Φ14	500	Φ6/200	220	13.40	61.85	56.94	61.85	56.94	77.65	36.4	1.36
	AS2C3(2)	200	200	320	30	0.351	240	4Φ14	500	Φ6/95	220	13.40	35.68	28.26	35.68	28.26	38.33	35.6	1.36
	AS2C5(2)	200	200	320	30	0.585	240	4Φ14	500	Φ6/95	220	13.40	47.61	42.62	47.61	42.62	47.17	10.7	1.11
	AS2G3(2)	200	200	320	30	0.4614	73	4Φ14	500	Φ6/95	220	13.40	32.14	36.60	32.14	36.60	48.42	32.3	1.32
	AS2G6(2)	200	200	320	30	0.9228	73	4Φ14	500	Φ6/95	220	13.40	48.69	43.01	48.69	43.01	64.92	50.9	1.51
	AS2G9(2)	200	200	320	30	1.3842	73	4Φ14	500	Φ6/95	220	13.40	65.68	54.95	65.68	54.95	78.06	42.1	1.42

**Table 3:** Geometrical and mechanical data of 11 RC columns and experimental–analytical results.

(**A**)																				
**Experiments**	**h** **(mm)**	**b** **(mm)**	**H** **(mm)**	**Long.**	**Stir.**	***n***	***t_FRP_*** **(mm)**	***f_c_*_0_** **(MPa)**	***f_cc, exp_*** **(MPa)**	***ε_cc_*** **(‰)**	***f_cu, exp_*** **(MPa)**	***ε_cu_*^1^** **(‰)**	* *f_cc_*, _*ana*l_ **(MPa)**	* *f_cu,_*_*anal*_ **(MPa)**	* *ε_FRP,_*_*mid,* stirrup_ **(‰)**	* *ε_FRP,mid_*, _between_ Stirrups_ **(‰)**	* *ε_max_/ε_min_*, _Middle_ **(‰)**	* *ε_FRP,Corner,Stirrup_* **(‰)**	* *ε_FRP,Corner,_*_between_ Stirrups_ **(‰)**	
BS1C1C	200	200	320	4Φ14	Φ8/200	1	0.117	25.5	37.34	2.38	31.74	7.95	33.29	34.99	4.805	12.307	2.561	8.211	7.634	
BS1C3C	200	200	320	4Φ14	Φ8/200	3	0.351	25.5	40.96	6.80	34.85	11.63	39.52	42.65	7.908	14.933	1.888	10.525	11.690	
BS1C5C	200	200	320	4Φ14	Φ8/200	5	0.585	25.5	55.35	10.13	53.60	10.83	53.10	53.10	7.239	12.452	1.720	8.967	10.148	
BS2C1C	200	200	320	4Φ14	Φ8/95	1	0.117	25.5	42.99	3.29	39.37	11.44	42.64	44.81	13.958	15.092	1.082	-^2^	7.091	
BS2C3C	200	200	320	4Φ14	Φ8/95	3	0.351	25.5	50.68	12.90	50.68	12.90	52.18	52.18	13.491	13.980	1.036	-^2^	9.179	
BS2C5C	200	200	320	4Φ14	Φ8/95	5	0.585	25.5	59.29	9.17	59.21	9.17	52.76	52.76	9.405	9.933	1.056	11.368	6.937	
LSR-R-1-3-10b	250	250	500	4Φ14	Φ8/200	3	0.495	10.83	26.60	78.00	26.60	78.00	21.31	21.31	4.151	6.418	1.546	-^2^	-^2^	
LSR-R-1-3-20b	250	250	500	4Φ14	Φ8/200	3	0.495	10.83	31.12	66.00	31.12	66.00	28.35	28.35	9.387	8.018	0.854	-^2^	-^2^	
LSR-R-1-3-40b	250	250	500	4Φ14	Φ8/200	3	0.495	10.83	41.17	68.00	48.07	68.00	42.27	42.27	12.531	12.142	0.969	-^2^	-^2^	
R2.0H2CL3	400	200	1000	8Φ16	Φ8/100	3	0.501	46.3	54.40	4.03	39.36	5.00	57.69	58.85	4.836	5.180	0.996	5.313	4.031	
R2.0H3CL4	400	200	1000	8Φ16	Φ8/100	4	0.668	46.3	57.80	3.93	39.36	6.00	59.48	58.76	5.923	5.906	1.003	4.778	6.394	
(**B**)																				
**Experiments**	* *ε_max_/ε_min_*, _Corner_ **(‰)**	* *ε_stirrup,Max_* **(‰)**		* *ε_long,M_*_i*d*_ **(‰)**	* *ε_long,Min_* **(‰)**	* Lateral Deformation (*Y* axis) of Long. Steel Bars at the Point of Stirrup		* Lateral Deformation (*X* axis) of Long. Steel Bars at the Point of Stirrup		* Lateral Deformation of Long. Steel Bars at the Point of Stirrup		* Lateral Deformation (*Y* axis) of Long. Steel Bars between Two Stirrups		* Lateral Deformation (*X* axis) of Long. Steel Bars between Two Stirrups		* Lateral Deformation of Long. Steel Bars between Two Stirrups	* Lateral Deformation of Middle Stirrup	***ε_cu,Pred._*** **(‰)**	**AAE (%)**	**AR**
BS1C1C	1.076	1.532		-11.436	−11.436	0.105		−0.111		0.153		0.576		−0.537		0.787	0.191	8.90	11.9	1.12
BS1C3C	0.900	1.992		−16.156	−16.156	0.144		−0.149		0.207		0.716		−0.697		0.999	0.010	11.59	0.3	1.00
BS1C5C	0.884	2.020		−14.730	−14.730	0.148		−0.141		0.205		0.590		−0.576		0.825	0.325	13.65	26.0	1.26
BS2C1C	2.754	7.565		−11.486	−22.760	0.419		−0.415		0.589		0.626		−0.621		0.882	0.927	9.30	18.7	0.81
BS2C3C	1.765	6.863		−12.110	−27.022	0.350		−0.353		0.497		0.572		−0.571		0.808	0.967	11.83	8.3	0.92
BS2C5C	1.639	4.461		−9.354	−18.632	0.271		−0.265		0.378		0.379		−0.377		0.534	0.672	13.83	50.8	1.51
LSR-R-1-3-10b	1.568	12.447		−191.940	−264.270	0.909		−0.094		0.914		−0.905		−4.179		4.275	4.899	66.50	2.2	0.98
LSR-R-1-3-20b	0.988	10.740		−83.820	−101.48	−0.238		0.302		0.384		−1.563		0.655		1.695	7.610	38.01	51.3	0.49
LSR-R-1-3-40b	0.752	15.350		−66.850	−104.86	0.071		−0.098		0.121		0.951		−0.918		1.322	6.788	47.41	28.2	0.72
R2.0H2CL3	1.396	2.243		−5.263	−9.067	0.357		0.125		0.378		0.416		0.173		0.451	0.446	5.60	12.0	1.12
R2.0H3CL4	0.760	2.600		−6.152	−10.560	−0.414		−0.146		0.439		−0.477		−0.200		0.517	−0.489	5.88	1.9	0.98

^1^ The finite element (FE) model analyses were stopped at the same stain as the experimental ones. ^2^ These values are larger than the strain of the material failure (15.5‰). * These values are the FE model analytical results.
